# Umbilical cord mesenchymal stem cell-derived exosomes inhibits fibrosis in human endometrial stromal cells via miR-140-3p/FOXP1/Smad axis

**DOI:** 10.1038/s41598-024-59093-5

**Published:** 2024-04-09

**Authors:** Mengling Song, Lijun Ma, Yongzhao Zhu, Huimin Gao, Rong Hu

**Affiliations:** 1 Department of Reproductive Medicine, General Hospital of Ningxia Medical University (The First Clinical Medical College of Ningxia Medical University), 804 Shengli Street, Xingqing Square, Yinchuan, 750004 Ningxia China; 2 Department of Human Anatomy, Histology and Embryology, School of Basic Medicine, Ningxia Medical University, Yinchuan, 750004 Ningxia China; 3 Institute of Medical Sciences, General Hospital of Ningxia Medical University, Yinchuan, 750004 Ningxia China; 4 General Hospital of Ningxia Medical University (the First Clinical Medical College of Ningxia Medical University), Yinchuan, 750004 Ningxia China

**Keywords:** Exosome, Endometrial fibrosis, MicroRNA, Umbilical cord mesenchymal stem cell, Transforming growth factor-β, Cell biology, Stem cells

## Abstract

Endometrial fibrosis is the histologic appearance of intrauterine adhesion (IUA). Emerging evidences demonstrated umbilical cord mesenchymal stem cell-derived exosomes (UCMSC-exo) could alleviate endometrial fibrosis. But the specific mechanism is not clear. In this study, we explored the effect of UCMSC-exo on endometrial fibrosis, and investigated the possible role of miR-140-3p/FOXP1/Smad axis in anti-fibrotic properties of UCMSC-exo. UCMSC-exo were isolated and identified. Transforming growth factor-β (TGF-β) was used to induce human endometrial stromal cell (HESC) fibrosis. Dual luciferase assay was performed to verify the relationship between miR-140-3p and FOXP1. The expressions of fibrotic markers, SIP1, and p-Smad2/p-Smad3 in HESCs stimulated with UCMSC-exo were detected by western blot. In addition, the effects of miR-140-3p mimic, miR-140-3p inhibitor and FOXP1 over-expression on endometrial fibrosis were assessed. The isolated UCMSC-exo had a typical cup-shaped morphology and could be internalized into HESCs. The expressions of fibrotic markers were significantly increased by TGF-β, which was reversed by UCMSC-exo. MiR-140-3p in UCMSC-exo ameliorated TGf-β-induced HESCs fibrosis. FOXP1 was identified as the direct target of miR-140-3p, which could inversely regulate miR-140-3p’s function on HESCs fibrosis. Furthermore, we demonstrated that miR-140-3p in UCMSC-exo regulated Smad signal pathway to exert the anti-fibrotic effect in HESCs. The anti-fibrotic effect of UCMSC-derived exosomes against HESC fibrosis was at least partially achieved by miR-140-3p/FOXP1/Smad axis.

## Introduction

Intrauterine adhesion (IUA) is a common endometrial disorder caused by trauma or infection. It is characterized by symptoms such as hypomenorrhea, amenorrhea, pelvic pain, infertility and recurrent abortion, etc.^1^. With the increase of uterine invasive procedures, the incidence of IUA has gradually increased year by year. Endometrial repair disorder and endometrial fibrosis have been regarded as the main mechanism of IUA formation^2^. Thus, finding a novel therapeutic strategy to reverse endometrial fibrosis has become an urgent problem to overcome IUA.

Mesenchymal stem cells (MSCs) are a type of multipotent somatic stem cells that can be derived from various fetal and adult tissues, such as bone marrow, umbilical cord, adipose tissue, and placenta^3^. These cells possess the capability to differentiate into different cell lineages, and have great potential in promoting cell growth and tissue repair, making them highly promising in the field of regenerative medicine^4^. For fibrotic diseases, MSCs derived from different sources have been validated to promote tissue repair and inhibit fibrosis in many organs including kidney, lung, heart and skin^5–7^.

Recent evidences have shown that the anti-fibrotic properties of MSCs are closely related to their paracrine mechanisms^8,9^. In which, exosomes are the most crucial paracrine factors of MSCs. And they are nano-sized particles consisting of a lipid bilayer that encapsulates biomolecules, including RNAs, DNAs, and soluble proteins^10,11^. Exosomes mediate intercellular communication by delivering biomolecules into target cells. Among the biomolecules carried by exosomes, microRNAs (miRNAs), small noncoding RNAs with 18–25 nucleotides in length, have attracted vast attention because of their important roles in regulating gene expression^8^. There are increasing evidences that exosomal miRNAs participated in the pathogenesis of organ fibrosis and tissue regeneration, and represent a promising treatment option for fibrotic diseases^8,12^. In endometrial fibrosis, several studies have reported that MSC-derived exosomal miRNAs exhibited an anti-fibrotic effect. For instance, Tan et al.^2^ found that exosomes derived from bone marrow MSC (BMSCs) released miR-29a to inhibit fibrosis during endometrial repair of IUA. Xiao et al.^13^ demonstrated that exosomes could metastasize BMSC-derived miR-340 in endometrial stromal cells to attenuate endometrial fibrosis. Compared to BMSCs, human umbilical cord-MSCs (UCMSCs) have advantages such as obtainment by a noninvasive procedure and easiness for culture. Li et al.^14^ reported that UCMSC-derived exosomes(UCMSC-exo) could reverse endometrial stromal cell fibrosis by regulating the miR-145-5p/ZEB2 axis.

miR-140-3p located on human chromosome 16q22.1 has been proved to regulate fibrogenesis in several models^15^. A study reported that up-regulating miR-140-3p could relieve renal fibrosis^15^. Furthermore, miR-140-3p has been demonstrated to be released by UCMSCs and reduce fibrosis in rheumatoid arthritis rats^16^. Zhijuan Hua et al. confirmed that metformin can alleviate subretinal fibrosis through promoting miR-140-3p expression, inhibiting LIN28B and the JNK/STAT3 pathway^17^. miR-140-3p can also suppress fibrogenesis in TGF-β1-induced HSC-T6 cells and may be contribute to remedy for liver fibrosis^18^. In addition, Forkhead box (FOX) protein P1 (FOXP1) is a transcription factor with various functions including enhancing fibrosis. For instance, Xu Shao et al. demonstrated that FOXP1 enhances fibrosis during endometriosis by up-regulating Wnt signaling activity^19^. Moreover, FOXP1 has capability to regulate the TGF-β1-endothelin-1 pathway to inhibit pathological cardiac fibrosis and hypertrophy^20^. In tissue fibrosis, TGF-β directly activates Smad signaling, triggering pro-fibrotic gene overexpression^21^. More importantly, previous studies have shown that miR-140-3p could directly interact with FOX family^22,23^. Thus, we hypothesized that the anti-fibrotic effect of UCMSC-derived exosomes was partially achieved by miR-140-3p/FOXP1/Smad axis.

In this study, we identified UCMSC-exo and explored the effect of UCMSC-exo on endometrial fibrosis. Furthermore, we investigated the possible role of miR-140-3p/FOXP1/Smad axis in anti-fibrotic properties of UCMSC-exo.

## Materials and methods

### Preparation of human UCMSCs (hUCMSCs)

Fresh umbilical cords were from the mothers who just gave birth, and informed consent was obtained from hospitalized mothers at General Hospital of Ningxia Medical University, and rapidly processed. The Ethics Committee of General Hospital of Ningxia Medical University approved this study (Ethical code: 2020-125). All experiments were performed in accordance with relevant guidelines and regulations. Repeatedly rinsed the cord several times with PBS and removed the blood vessels and the outer membrane of the umbilical cord. Then, the cords were cut into small pieces and were cultured in Nuwacell ncMission hMSC Medium (Nuwacell, China) supplemented with 1 × GlutaMAX™ and 50 μg/ml gentamycin (Thermofisher scientific, Carlsbad, CA, USA). All cultures were maintained at 37 °C in a humidified incubator with 5% CO_2_. The medium was changed every 3 days and the cells were passaged after detachment with Recombinant Trypsin EDTA Solution (Biological Industries, Israel). All experiments used the less than 5th passages hUCMSCs.

### Flow cytometry analysis

Surface phenotype analysis of hUCMSCs was evaluated by flow cytometry. Briefly, hUCMSCs were detached and incubated with fluorescein-labeled antibodies for 30 min at 4 ℃. The tested antibodies included CD73, CD90, CD105, CD14, CD34, CD45, and HLA-DR (BD Biosciences, USA). Following extensive washing, the cells of each group were resuspended in 400 μL of PBS and detected by flow cytometry (BD Biosciences).

### Multilineage differentiation assay in vitro

To evaluate adipogenic and osteogenic differentiation potential, hUCMSCs were incubated with adipogenic and osteogenic differentiation medium (BI Biological Industries, Israel) for 21 days and the medium was changed every 3 days. Adipogenic and osteogenesis were identified by adipogenic and osteogenic staining kit (BI Biological Industries, Israel) respectively. To assess cartilage differentiation potential, a cell solution of 1.6 × 10^7^ hUCMSCs /mL to form a pellet by gravity. The pellet cells were cultured with cartilage differentiation medium (BI Biological Industries, Israel) for 21 days and the medium was changed twice a week. Chondrocytes were detected by a cartilage staining kit (BI Biological Industries, Israel). All the measurements were tested three times. All images were viewed under a bright field of view using an inverted phase contrast microscope (Olympus, Japan).

### Cell culture

Human endometrial stromal cell (HESC) line was purchased from the American Type Culture Collection (ATCC CRL-4003) and was cultured in DMEM/F12 medium supplemented with 10% charcoal-stripped fetal calf serum and antibiotics (BI Biological Industries, Israel). All cells were cultured at 37 °C in a 5% CO_2_ humidified incubator. At about 80% confluence, the cells were passaged after detachment with Recombinant Trypsin EDTA Solution.

### Isolation and identification of UCMSC-exo

Exosomes were extracted from the cell culture medium of UCMSCs using a VEX exosome isolation reagent (Vazyme, Nanjing, China) as previously described^24^. Briefly, the culture supernatant was centrifuged at 4 °C 3000*g* for 30 min to remove cells and passed through a 0.22 μm filter (Millipore, USA) to eliminate cell debris and dead cells. Subsequently, the VEX Exosome Isolation Reagent was added into the supernatant and the mixture was vortexed and incubated at 4 °C for up to 16 h. Then, the mixture was centrifuged at 10,000*g* 4 °C for 30 min to precipitate exosome pellets. Finally, the pelleted exosomes were resuspended in PBS and stored at − 80 °C.

The total protein concentration of exosomes was detected using a bicinchoninic acid (BCA) protein assay kit (Beyotime, Shanghai, China). The expression levels of exosome-specific biomarkers CD9 (ab236630, Abcam, Cambridge, USA) and TSG101 (ab133586, Abcam) were analyzed by western blot. Nanoparticle tracking analysis (NTA) with ZetaView^®^ (Particle Metrix, Germany) was emplpyed to quantify the size of exosomes. The exosome morphology was captured by a transmission electron microscopy (TEM, HT-7700, Hitachi, Japan). To detect the internalization of exosomes in HESCs, the purified exosomes were labeled with a red fluorescence dye PKH26 (SigmaAldrich, St. Louis, USA) according to the manufacturer's instructions. HESCs and PKH26 labeled-exosomes were co-cultured for 24 h and fixed with 4% paraformaldehyde. After the nuclear was counterstained with DAPI, the images were observed by Olympus FSX100 microscope (Olympus, Japan).

### Treatment and cell transfection

Based on existing research^14,25^, using 50 ng/ml TGF-β dealing with HESC to induce fibrosis. It has been suggested that 20 µg/ml of UCMSC-exo could significantly enhance the viability of HESCs^26^. Thus, the dose of 20 µg/ml was selected in this study to determine the anti-fibrotic properties of UCMSC-exo.

Expression vectors encoding FOXP1 were constructed and inserted into the pcDNA3.1 vector (oeFOXP1), while the empty pcDNA3.1 vectors were used as negative control (oeNC). The GenePharma Company (Shanghai, China) was responsible for the creation of the two vectors. MiR-140-3p inhibitor (anti-miR-140-3p), miR-140-3p mimic and their corresponding negative controls were constructed by Guangzhou RiboBio Co., Ltd. Using Lipofectamine 3000 reagent (Invitrogen), oeFOXP1 and oeNC were transfected in HESCs to overexpress FOXP1, anti-miR-140-3p, miR-140-3p mimics and their controls were transfected in UCMSCs.

### Dual luciferase reporter assay

To verify whether miR-140-3p was targeted at FOXP1, luciferase reporter assay was performed. Clone the FOXP1 sequence, including wild type (WT) (GGCTTTGGTCAGCATTTTTCATTTAAAGAAAAGTAACACTCCCATCCACTCATAAGCTTGGTACAAAAACTTCTCTGGCAGTTACTTTTGAAGCTTCACTCTGCTTTCTGTATAAAGGGCAGT**CTGTGGT**CACGCAAGACTTTAAAAAAA) and mutant type (MUT) (GGCTTTGGTCAGCATTTTTCATTTAAAGAAAAGTAACACTCCCATCCACTCATAAGCTTGGTACAAAAACTTCTCTGGCAGTTACTTTTGAAGCTTCACTCTGCTTTCTGTATAAAGGGCAGT**TCACAAC**CACGCAAGACTTTAAAAAAA) of miR-140-3p binding sites (3’UTR), downstream of the firefly luciferase gene in the pGL3 luciferase reporter vectors. Using the Lipofectamine 3000 reagent (Invitrogen), co-transfect the FOXP1 wild type vector (FOXP1-3’UTR-WT) and mutant type vector (FOXP1-3’UTR-MUT) into HESCs with miR-140-3p mimic or mimic NC. After transfection of 48 h, the luciferase activity was measured using the Dual-Glo Luciferase Assay System.

### qRT-PCR

Total RNA was extracted from exosomes and cells using the TRIzol reagent (Invitrogen) and reversely transcribed into cDNA by cDNA Synthesis Kit (TaKaRa Bio, USA). An ABI StepOnePlus™ System (Applied Biosystems, USA) was utilized to perform qRT-PCR. The relative expression of miRNA and mRNA were normalized to U6 and β-act, in respectively. The following primers were presented:miR-140-3p,forward 5’-CAGTGCTGTACCACAGGGTAGA-3’ and reverse 5’-TATCCTTGTTCACGACTCCTTCAC-3’;FOXP1,forward 5’-ATGGAGCATACCAACAGCAACG-3’ and reverse 5’-ACTGTGGTTGGCTGTTGTCAC-3’. The data were calculated using the 2^–△△Ct^ method.

### Western blotting

The proteins extracted from cells were separated and transferred onto PVDF membranes (Millipore, USA). After blocking with 5% skimmed milk, the membranes were incubated with antibodies CD9 (1:1000, ab236630, Abcam), TSG101 (1:1000, ab133586, Abcam), α-smooth muscle actin [α-SMA, 1:1000, #19245, Cell Signaling Technology (CST), Danvers, USA], collagen type I α1 chain (COL1A1, 1:1000, #72026, CST), connective tissue growth factor (CTGF, 1:1000, #86641, CST), FOXP1 (1:1000, #4402, CST), phosphor(p)-Smad2 (1:1000, #18338, CST), p-Smad3 (1:1000, #9520, CST), SMAD2/3 (1:1000, #8685, CST), and SMAD interacting protein 1 (SIP1, 1:1000, #97885, CST). After that, the membranes were incubated with HRP-linked secondary antibody. ECL (Millipore) solution was used to observe the protein band. Relative protein expression was quantified by BioImaging Systems.

### Statistical analysis

All data were analyzed by SPSS 22.0. The measurement data were described as mean ± Standard Deviation (SD). The differences among groups were evaluated by a Student’s *t* test (for 2 groups) or ANOVA (for more than 2 groups) followed by a LSD post hoc test. *P* < 0.05 was considered statistically significant.

### Ethical approval

The study was approved by the Medical Ethics Committee of General Hospital of Ningxia Medical University (No. 2020-125).

### Consent to participate

All authors agree to participate in this project and research.

## Results

### The characterization of hUCMSC

**Figure 1:**
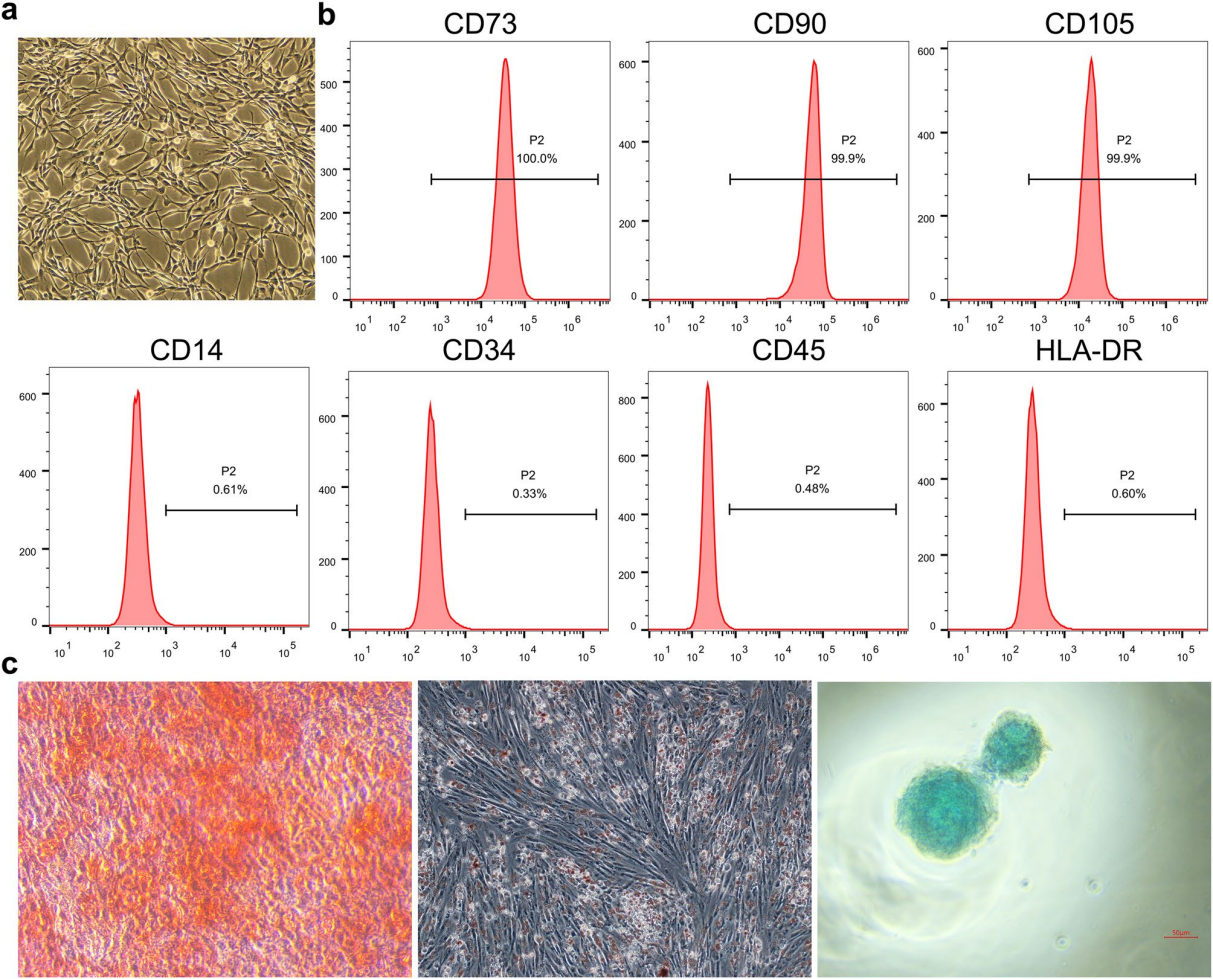
The characterization of UCMSC. (**a**) Morphology of UCMSCs under light field microscope, scale bars = 100 μm. (**b**) UCMSCs were highly positive for mesenchymal stem cells surface markers, including CD73, CD90, and CD105, but negative for CD14, CD34, CD45 and HLA-DR by flow cytometry analysis. (**c**) The multipotency of UCMSCs was detected via osteogenic, adipogenic, and chondrogenic differentiation using alizarin red staining, oil red O staining and alcian blue staining.

The isolated hUCMSC demonstrated a fibroblast-like, spindle-shaped morphology (Fig. 1a). UCMSCs were highly positive for mesenchymal stem cells surface markers, including CD73, CD90, and CD105, but negative for CD14, CD34, CD45 and HLA-DR by flow cytometry analysis (Fig. 1b). The multipotency of hUCMSCs was detected via osteogenic, adipogenic, and chondrogenic differentiation. Osteogenic differentiation indicated the presence of calcium deposition, adipogenic differentiation exhibited lipid droplets in the cytoplasm, and chondrogenic differentiation showed typical glycosaminoglycans in cartilage, as evidenced by alizarin red staining, oil red O staining and alcian blue staining, respectively (Fig. 1c). These features indicate that the isolated cells were consistent with typical UCMSC characteristics.

### The characterization of UCMSC-exo

**Figure 2:**
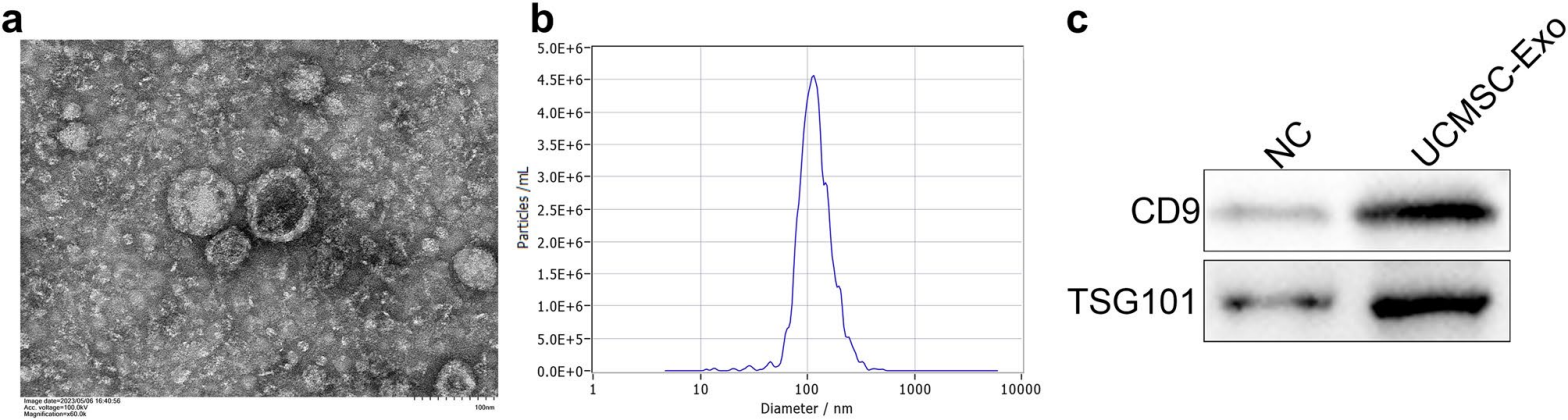
The characterization of UCMSC-exo. (**a**) The morphology of UCMSC-derived exosomes was detected by a transmission electron microscopy. (**b**) The main diameter of the exosomes was determined by nanoparticle tracking analysis. (**c**) The expression of exosomal marker proteins in UCMSC-derived exosomes was determined by western blotting analysis.

Then, we collected cell-conditioned medium of UCMSCs and extracted exosomes. As shown in Fig. 2a, UCMSC-derived particles presented a typical cup-shaped morphology by TEM. NTA analysis identified the mean diameter of the particles was 125.7 nm (Fig. 2b). In addition, western blotting analysis revealed that the exosomal marker proteins (CD9 and TSG101) were abundant in the UCMSC-derived particles (Fig. 2c). The above results indicated that the particles extracted from UCMSC were exosomes.

### UCMSC-exo reversed TGF-β-induced fibrosis of HESCs

**Figure 3:**
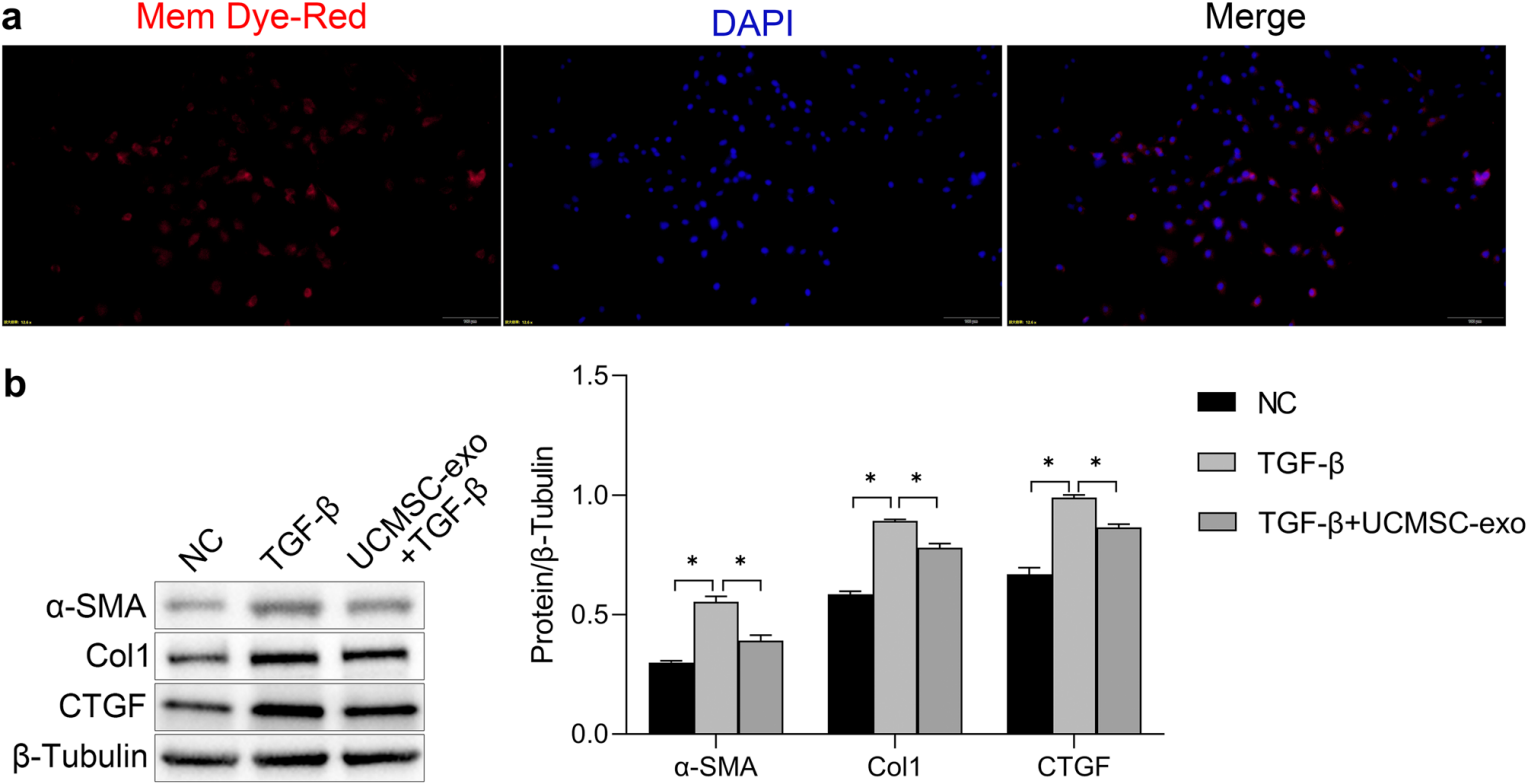
UCMSC-exo reversed TGFβ-induced fibrosis in HESC cells in human endometrial stromal cells (HESCs). (**a**) Representative images of the internalization of PKH-26-labeled UCMSC-exo into HESCs, scale bars = 100 μm. (**b**) HESCs were treated with 50 ng/ml TGF-β or 20 µg/ml UCMSC-exo combined with 50 ng/ml TGF-β for 48 h, the fibrosis-related markers including α-SMA, COL1A1 and CTGF were detected by western blotting.

To evaluate the impact of UCMSC-exo on HESCs, we firstly detected whether UCMSC-exo could be internalized into HESCs. As shown in Fig. 3a, PKH-26-labled UCMSC-exo were traced in the perinuclear and nuclear regions of HESCs. Then, we detected the role of UCMSC-exo on TGF-β-induced fibrosis. One HESCs was treated with 50 ng/ml TGF-β for 48 h, and another HESCs meanwhile was adressed by 20 µg/ml UCMSC-exo in combination with 50 ng/ml TGF-β for 48 h, the fibrosis-related markers including α-SMA, COL1A1 and CTGF were detected by western blotting. Results showed a significant increase in protein levels of α-SMA, COL1A1 and CTGF in TGF-β treated group compared to the control group (*P* < 0.05). However, UCMSC-exo reversed TGF-β-inducing HESC fibrosis. The expression of fibrosis-related markers were significantly lower in UCMSC-exo + TGF-β group compared to the TGF-β group (Fig. 3b).

### MiR-140-3p in UCMSC-exo exerted an anti-fibrotic effect in HESCs

**Figure 4:**
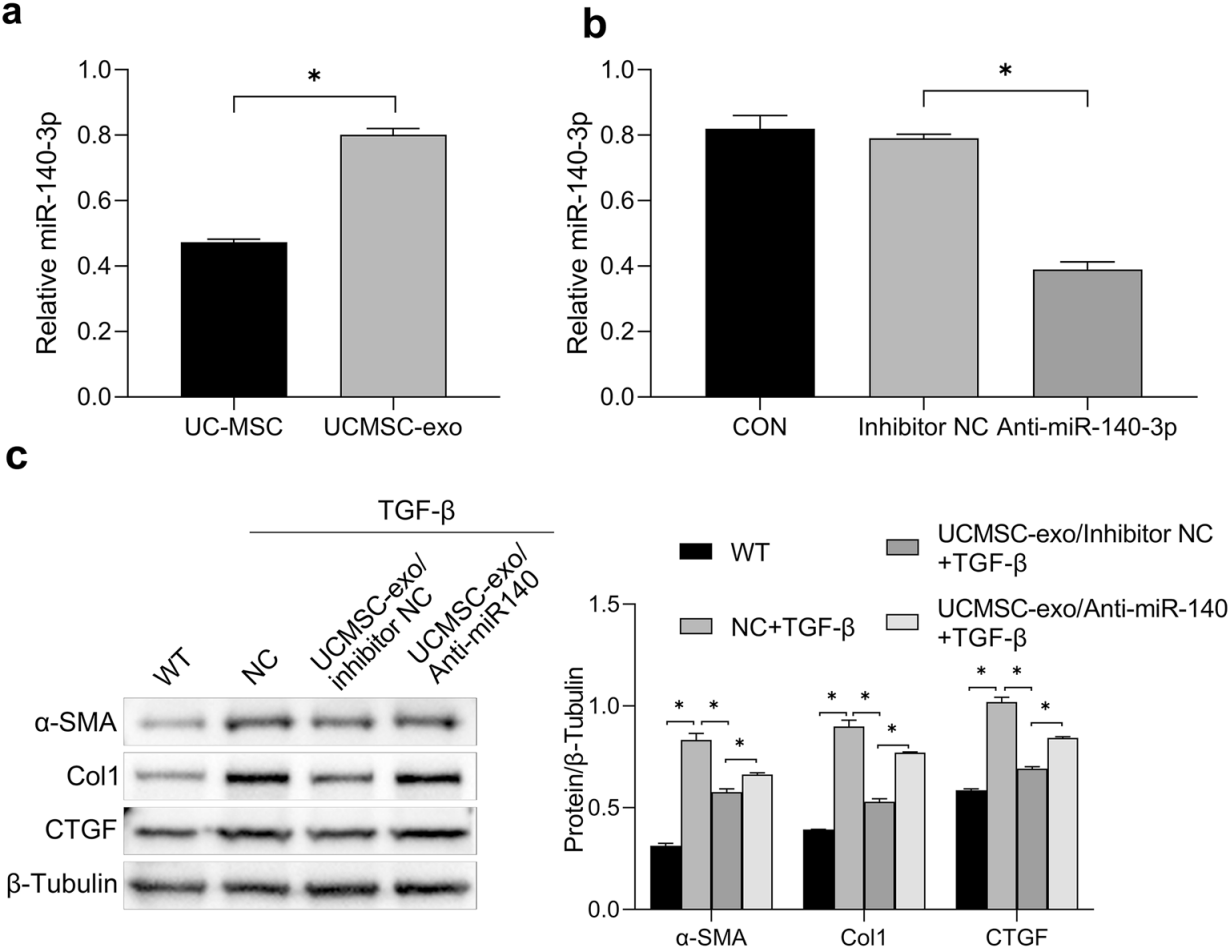
MiR-140-3p in UCMSC-exo exerted anti-fibrotic effect in human endometrial stromal cells (HESCs). (**a**) The expression of miR-140-3p in UCMSC-exo was detected by RT-qPCR. (**b**) The expression of miR-140-3p in UCMSC-exo transfected with miR-140-3p inhibitor was determined by RT-qPCR. (**c**) MiR-140-3p inhibitor and inhibitor NC were transfected into UCMSCs and the exosomes were collected. Western blot analysis detected the level of fibrosis-related markers under the treatment of TGF-β and/or UCMSC-exo.

MiR-140-3p has been demonstrated to be released by UCMSCs exosome^17^. Interestingly, our results showed that the expression of miR-140-3p in UCMSC-exo was much higher than that of UCMSC (Fig. 4a). To verify whether miR-140-3p participated in the anti-fibrotic effect of UCMSC-exo on HESCs, we transfected miR-140-3p inhibitor into UCMSCs to silence miR-140-3p, and collected cell-conditioned medium to isolate exosomes. The qRT-PCR results showed that the expression of miR-140-3p was decreased in UCMSC-exo transfected with miR-140-3p inhibitor (UCMSC-exo/anti-miR-140-3p), with statistical differences compared to the negative control (UCMSC-exo/anti-miR-140-3p NC, *P* < 0.05, Fig. 4b). Then, HESCs were induced by 50 ng/ml TGF-β and treated with 20 µg/ml UCMSC-exo/anti-miR-140-3p or UCMSC-exo/anti-miR-140-3p NC for 48 h. The protein levels of fibrosis-related markers were detected by western blotting. We found that UCMSC-exo/anti-miR-140-3p NC reduced the expressions of fibrosis-related markers like UCMSC-exo. More importantly, the expressions of α-SMA, COL1A1 and CTGF in HESCs exposure to UCMSC-exo/anti-miR-140-3p were enhanced compared to UCMSC-exo/anti-miR-140-3p NC group, with significantly statistical differences between the two groups (*P* < 0.05, Fig. 4c). These findings confirmed that the anti-fibrotic effect of UCMSC-exo on HESCs was strongly associated with miR-140-3p.

### MiR-140-3p directly targeted FOXP1 to inhibit HESC fibrosis

**Figure 5:**
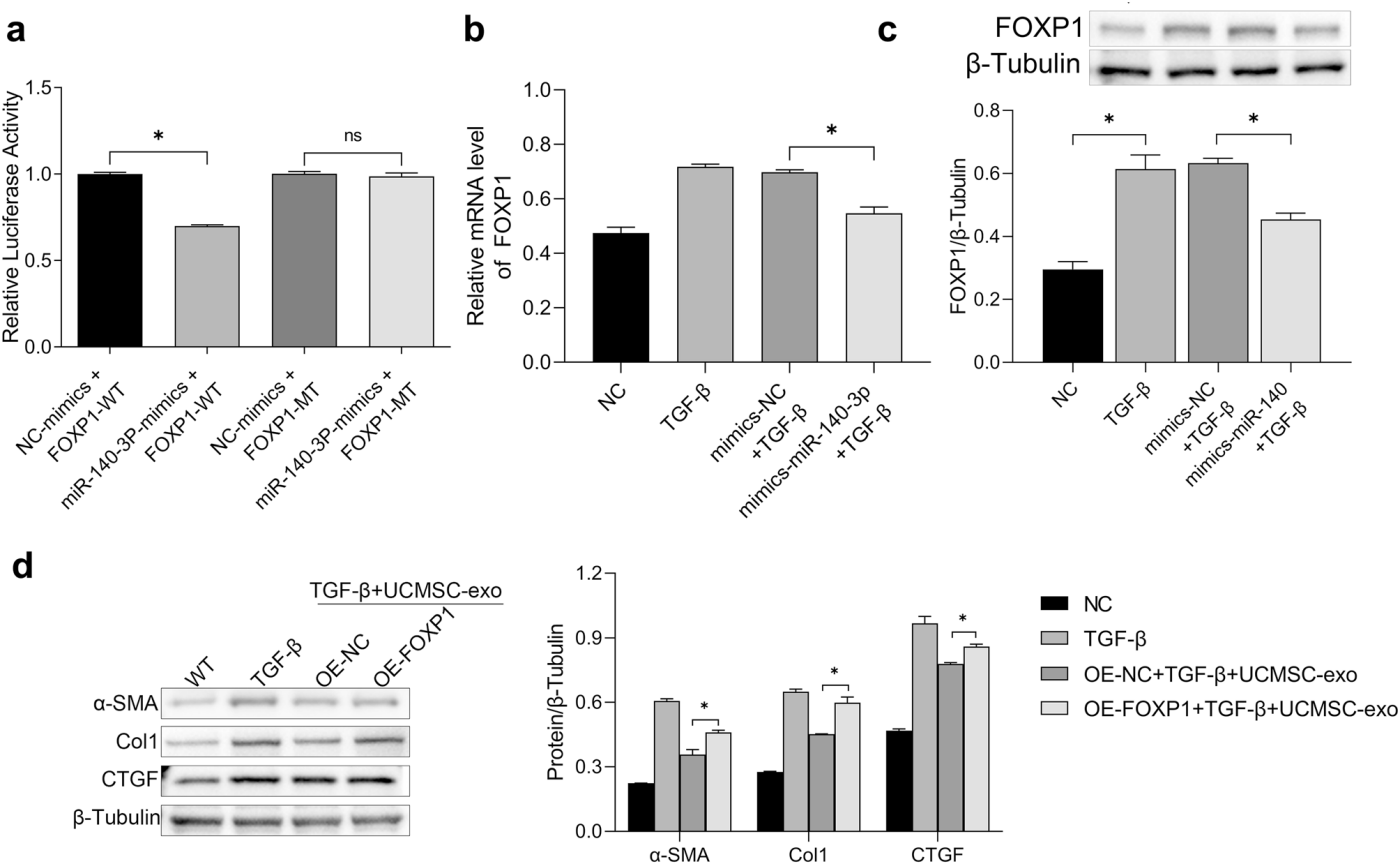
MiR-140-3p directly targeted FOXP1 to inhibit HESC fibrosis. (**a**) The luciferase reporter assay was used to detect whether miR-140-3p was targeted at FOXP1. (**b**) HESCs were transfected with miR-140-3p mimics or mimics NC, the expressions of FOXP1 were detected at RNA levels. (**c**) HESCs were transfected with miR-140-3p mimics or mimics NC, the expressions of FOXP1 were detected at protein levels. (**d**) OE-FOXP1 and OE-NC were transfected into HESCs. Western blot analysis detected the level of fibrosis-related markers under the treatment of TGF-β and/or UCMSC-exo.

MiRNA can regulate the expression of downstream target genes at the translational and post-translational level by binding to 3’UTR of the corresponding mRNA. We utilized Target Scan database and the luciferase reporter assay to predict and verify the regulatory relationship between miR-140-3p and FOXP1. The relative luciferase activity was notably suppressed in HESC co-transfected with miR-140-3p mimics and FOXP1-3’UTR-WT, whereas that in HESC co-transfected with miR-140-3p mimics and FOXP1-3’UTR-MUT did not appear to be suppressed (Fig. 5a). Subsequently, HESCs were transfected with miR-140-3p mimics or miR-140-3p NC, the expressions of FOXP1 were detected at gene and protein levels. qRT-PCR and western blotting analysis indicated that the expressions of FOXP1 in miR-140-3p mimics group were significantly lower than those in miR-140-3p NC group (*p* < 0.05, Fig. 5b,c). The above results suggest that FOXP1 was the direct target of miR-140-3p. We further detected the role of FOXP1 in HESC fibrosis. HESCs were transfected with oeFOXP1 and oeNC. Then, HESCs and transfected cells were treated with 50 ng/ml TGF-β1 and/or 20 µg/ml UCMSC-exo. Comparing to HESCs treated with TGF-β, transfected cells treated with TGF-β and UCMSC-exo had much lower expressions of fibrosis-related markers (Fig. 5d). Furthermore, the expressions of α-SMA, COL1A1 and CTGF were increased in oeFOXP1-transfected HESCs compared oeNC (*P* < 0.05, Fig. 5d). The aforementioned data suggested that miR-140-3p inhibited HESC fibrosis by targeting FOXP1.

### Exosomal miR-140-3p targeted FOXP1 to regulate TGF-β/Smad pathway

**Figure 6:**
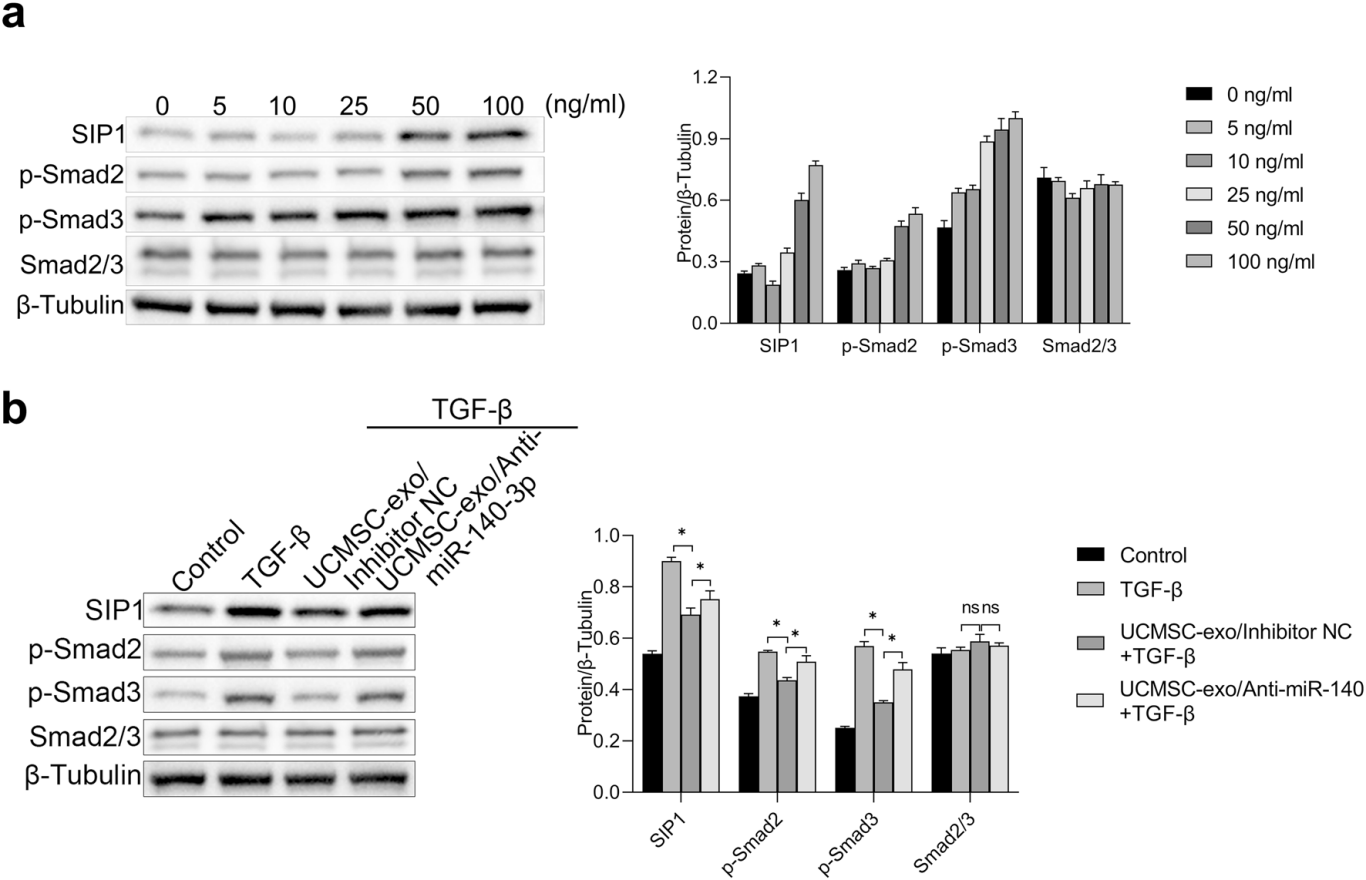
Exosomal miR-140-3p targeted FOXP1 to regulate TGF-β/Smad pathway. (**a**) HESCs were treated with different concentrations of TGF-β (0, 5, 10, 25, 50 and 100 ng/ml) and the expressions of proteins in Smad pathway were detected by western blot. (**b**) UCMSCs were transfected with miR-140-3p inhibitor or negative control and the exosomes were collected. The expressions of proteins in Smad pathway under the treatment of TGF-β and/or UCMSC-exo were detected by western blot.

It is well known to all, the Smad signaling pathway plays a indispensable role in nearly almost all fibrotic diseases. Consequently, we investigated the effect of exosomal miR-140-3p and TGF-β on the Smad pathway in HESCs. First, HESCs were exposed to varing levels of TGF-β (0, 5, 10, 25, 50 and 100 ng/ml) and the expressions of proteins in Smad pathway were detected by western blot. The results showed that the expressions of SIP1, p-Smad2 and p-Smad3 exhibited a dose-dependently increase with TGF-β treatment, whereas the levels of total Smad2/3 were unchanged (Fig. 6a). Our findings indicated that TGF-β could activate the Smad signaling pathway.

Then, UCMSCs were transfected with miR-140-3p inhibitor or negative control and the exosomes (UCMSC-exo/anti-miR-140-3p and UCMSC-exo/anti-miR-140-3p NC) were collected. The HESCs were subjected to the subsequent treatments for 48 h: (a) 50 ng/ml TGF-β, (b) 20 µg/ml UCMSC-exo/anti-miR-140-3p + 50 ng/ml TGF-β, (c) 20 µg/ml UCMSC-exo/anti-miR-140-3p NC + 50 ng/ml TGF-β, and (d) control. As expected, TGF-β caused an upsurge in the levels of SIP1, p-Smad2, p-Smad3 compared with the control (Fig. 6b).However,UCMSC-exo (UCMSC-exo/anti-miR-140-3p NC) reversed the effect of TGF-β on Smad signaling. More importantly, knocking down miR-140-3p could attenuate the inhibitory effect of UCMSC-exo on Smad signaling. The expressions of SIP1, p-Smad2, and p-Smad3 in HESCs exposure to UCMSC-exo/anti-miR-140-3p + TGF-β were significantly enhanced compared to UCMSC-exo/anti-miR-140-3p NC + TGF-β group, with significantly statistical differences between two groups (*P* < 0.05, Fig. 6b). These findings indicated that miR-140-3p in UCMSC-exo modulated the Smad signaling pathway, resulting in an anti-fibrotic effect in HESCs.

## Discussion

IUA is a prevalent endometrial disorder that significantly threaten women's physical and mental health^27^. Symptoms of this condition include hypomenorrhea, amenorrhea, pelvic pain, infertility and recurrent abortion, etc. Hysteroscopic adhesiolysis is a recommended approach for IUA^14^. However, the recurrence rate of adhesions after hysteroscopic adhesiolysis can reach to as high as 62.5%^28^. Thus, it is crucial to discover a novel therapeutic strategy to overcome IUA.

MSCs with different origins have been shown to be effective in inhibiting fibrosis in many organs^5–7^. Exosomes derived from MSCs exhibited a strong correlation with the anti-fibrotic properties of MSCs. MSC-exos can block myofibroblast differentiation and facilitate wound healing, emerging as important agents in fibrosis progression^29,30^. Consistent with the aforementioned discoveries, our results indicated that the expressions of fibrotic marker protein, α-SMA, COL1A1 and CTGF, were significantly decreased by co-cultured with UCMSC-exo in TGF-β mediated pathological microenvironment. These suggested that UCMSC-exo has capable to reverse HESC fibrosis and provided a promising strategy to alleviate clinical symptoms of IUA.

MSC-exos exhibited their regulatory function by transferring diverse biomolecules into target cells^8^. miRNAs are the important biomolecules carried by MSC-exos. Previously, the potential role of exosomal miRNAs in the treatment of endometrial fibrosis and IUA has been investigated. Exosomal miR-29a^2^ and miR-340^13^ from BMSCs, and miR-202-3p^31^, miR-145-5p^14^ and miR-543^32^ from UCMSCs have been reported to inhibit endometrial fibrosis in IUA by targeting relevant genes. MiR-140-3p, a miRNA which may be released by UCMSCs exosome, can help to reduce renal fibrosis^15^. It is a promising miRNA to treat fibrotic diseases. Our data suggested that miR-140-3p in UCMSC-exo, like other exosomal miRNA, had the capacity to inhibit fibrotic in HESCs. Our results showed that knocking down miR-140-3p in UCMSC-exo could reverse the inhibitory effect of UCMSC-exo on HESC fibrosis. Combining our data and those from other teams^14,31,32^, we could draw the conclusion that different exosomal miRNAs from UCMSCs could regulate endometrial fibrosis by different ways. Nevertheless, it has yet been conducted to determine which exosomal miRNA has the most significant impact.

It has been previously established that miR-140-3p has a direct interaction with the FOX family^22,23^. In this study, we investigated if miR-140-3p could target FOX family to exhibit anti-fibrotic role in the formation of endometrial fibrosis. Our research found that FOXP1 was the direct target of miR-140-3p. FOXP1 plays an essential role in the development of fibrosis. The importance of FOXP1 in fibrosis disease have been demonstrated by numerous studies^19,20^. FOXP1 can directly target TGF-β related signals and promoting TGF-β-inducing fibrosis^20,33^. In the present study, we found that using oeFOXP1 to overexpress FOXP1 in HESCs could reverse the effect of UCMSC-exo on HESC fibrosis, suggesting that miR-140-3p specifically targeted FOXP1 to inhibit HESC fibrosis (Supplementary Information).

Excessive data have demonstrated that dysregulation of TGF-β/Smad pathway was an important pathogenic mechanism in tissue fibrosis^21^. Two primary downstream effectors, Smad2 and Smad3 are responsible for promoting TGF-β-mediated fibrosis^21^. TGF-β activates the TGF-β receptor type I kinase, resulting in phosphorylation of SMAD2 and SMAD3. Subsequently, p-SMAD2 and p-SMAD3 form oligomeric complexes with SMAD4, which could regulate the transcription of ECM related genes to induce fibrosis^34^. SIP1, also known as Zinc finger E-box binding homeobox 2 (ZEB2), is a transcription factor that mediates epithelial‐mesenchymal transition process. Previous studies have shown that SIP1 functions as a DNA-binding transcriptional effector to interact with activated Smads, regulating TGF-β/Smad signaling pathway^35,36^. SIP1 regulates epithelial‐mesenchymal transition and fibroblast differentiation. Studies have indicated that miRNAs can be utilized to target SIP1, which has been linked to the prevention of fibrosis diseases^14,37,38^. Our findings corroborate previous studies showed that TGF-β activated the Smad signaling pathway and increased the expressions of SIP1, p-Smad2 and p-Smad3. Nevertheless, UCMSC-exo reversed the effect of TGF-β on Smad signal, which could be attenuated by the knockdown of miR-140-3p in UCMSC-exo. These findings indicated that miR-140-3p in UCMSC-exo modulated Smad signaling pathway to exert the anti-fibrotic effect in HESCs. Thus, we drew the preliminary conclusion that the anti-fibrotic effect of UCMSC-derived exosomes was at least partially achieved by miR-140-3p/FOXP1/Smad axis.

## Conclusion

In this study, UCMSC-exo were demonstrated to inhibit TGF-β-inducing HESC fibrosis, playing an anti-fibrotic role in the formation of endometrial fibrosis. In addition, UCMSC-exo were found to exert their anti-fibrotic function against HESC fibrosis through delivering miR-140-3p to target FOXP1, subsequently regulating TGF-β/Smad pathway. Taken together, our findings indicated that the anti-fibrotic effect of UCMSC-derived exosomes against HESC fibrosis was at least partially achieved by miR-140-3p/FOXP1/Smad axis.

### Supplementary Information

Supplementary Information.

## Data Availability

On reasonable request, the corresponding author will provide the datasets used and/or analyzed during the current work.
