# Supplementary material for: Umbilical cord mesenchymal stem cell-derived exosomes inhibits fibrosis in human endometrial stromal cells via miR-140-3p/FOXP1/Smad axis
Source: Sci Rep. 2024 Apr 9;14:8321. doi: 10.1038/s41598-024-59093-5 (PMC11004014; doi:10.1038/s41598-024-59093-5)
Supplement: Supplementary file 1 — Supplementary Information. [file 41598_2024_59093_MOESM1_ESM.pdf]

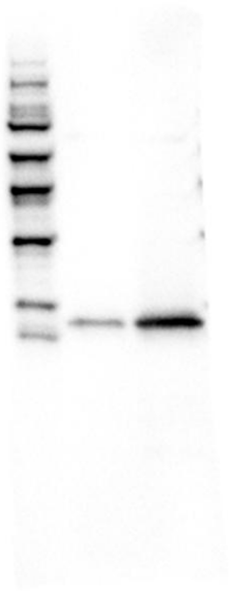

Fig2c CD9

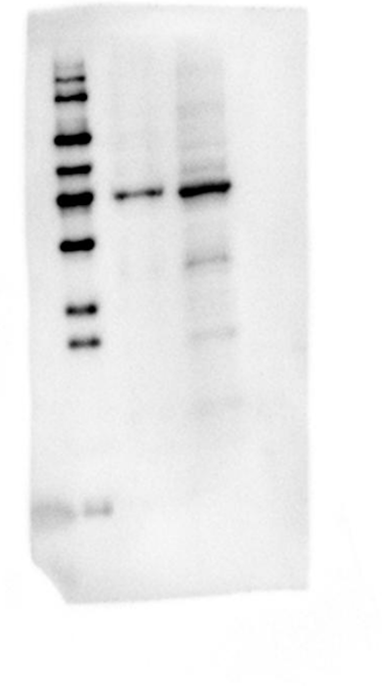

Fig2c TSG101

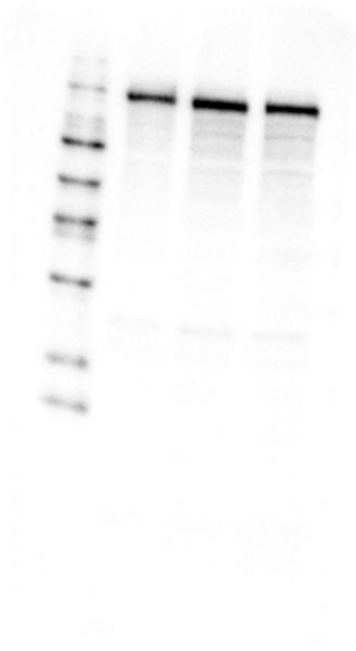

Fig3b Col1

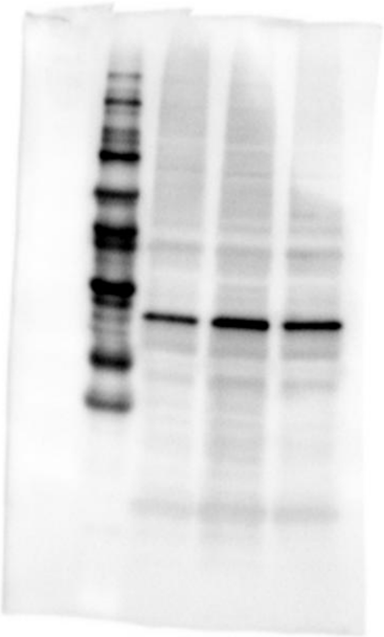

Fig3b CTGF

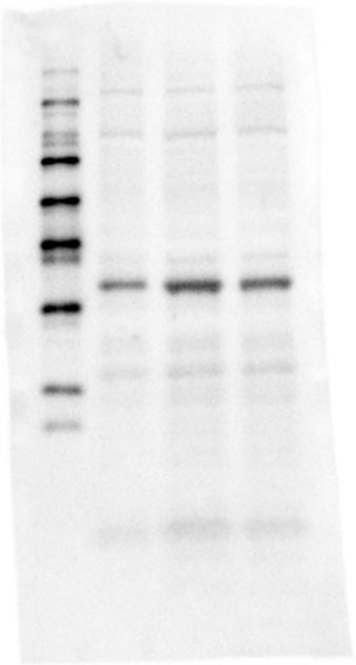

Fig3b α-SMA

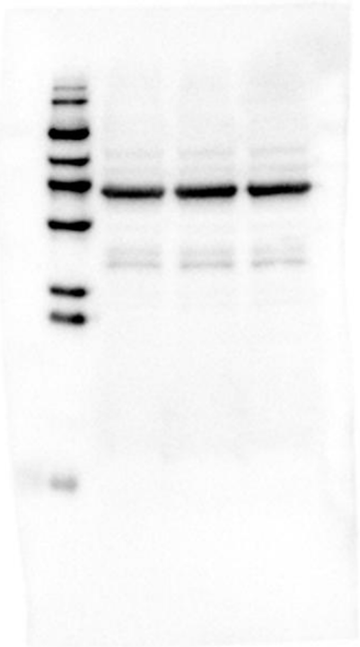

Fig3b β-Tubulin

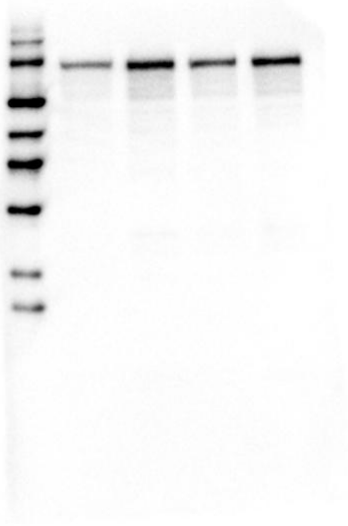

Fig4c Col1

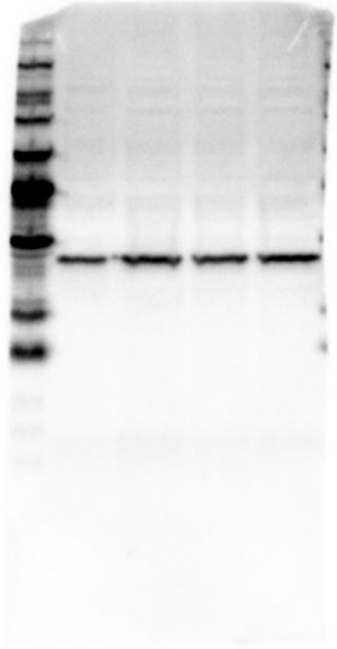

Fig4c CTGF

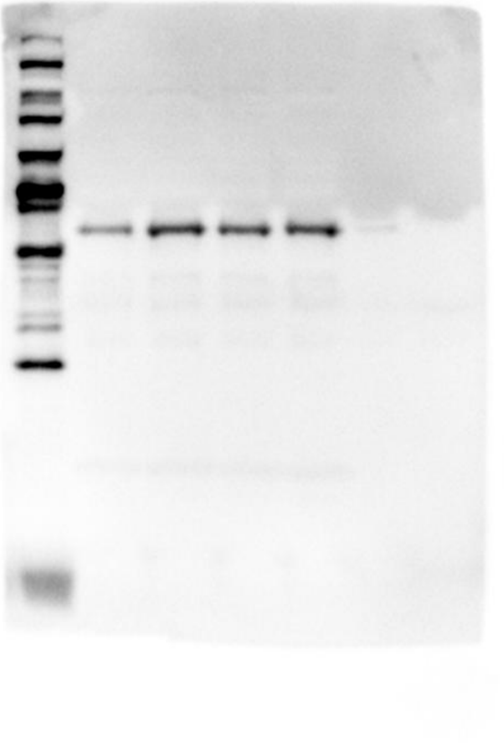

Fig4c α-SMA

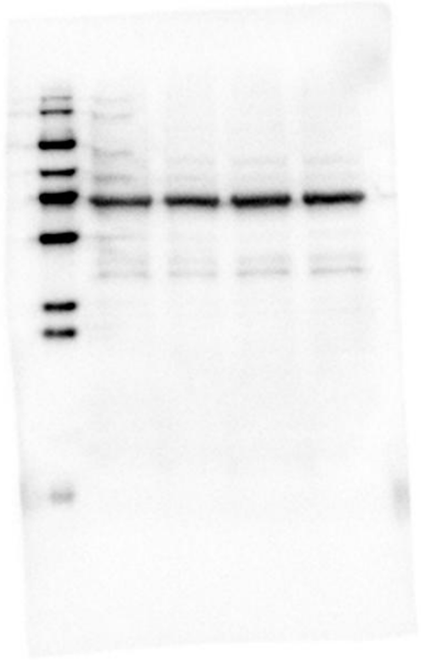

Fig4c β-Tubulin

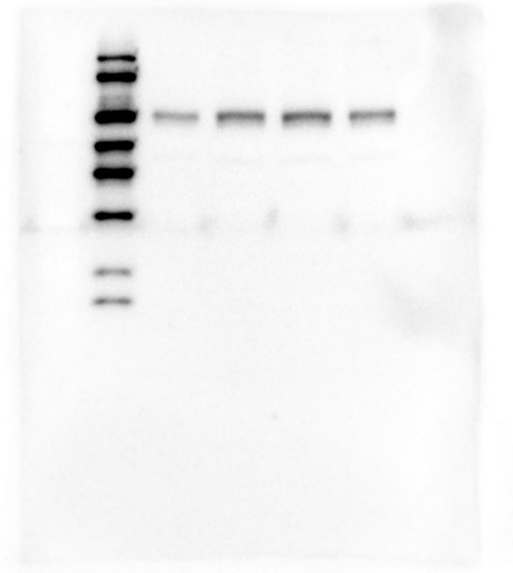

Fig5c FOXP1

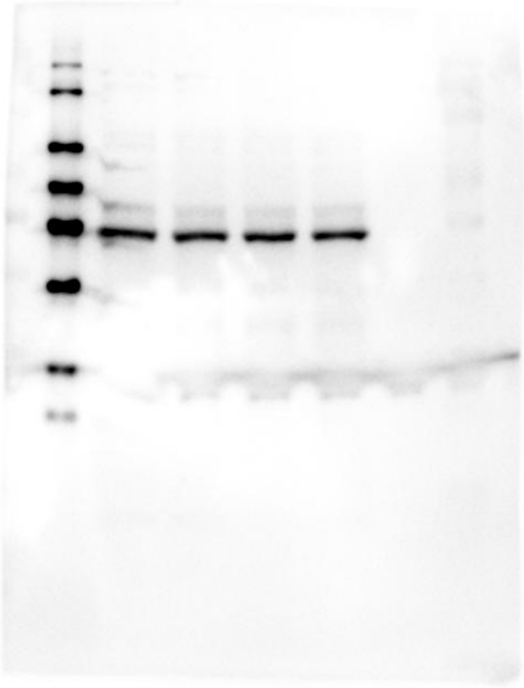

Fig5c β-Tubulin

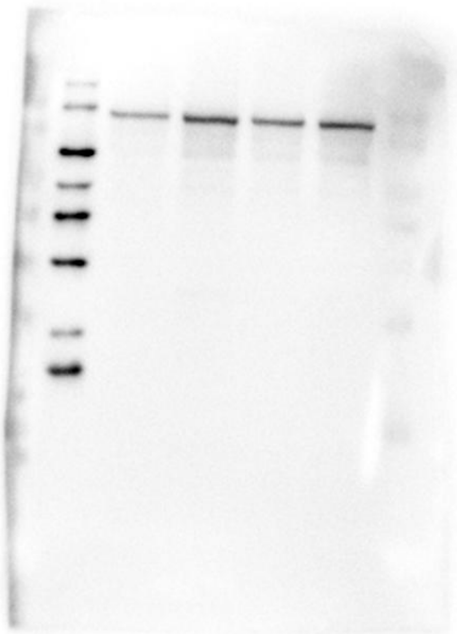

Fig5d Col1

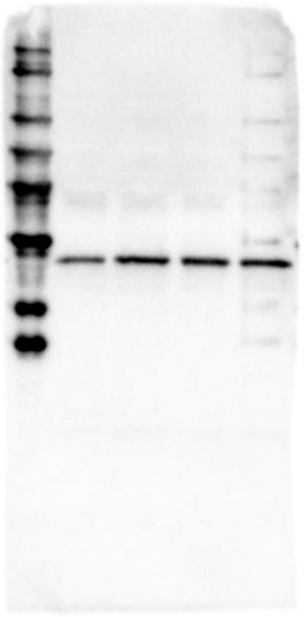

Fig5d CTGF

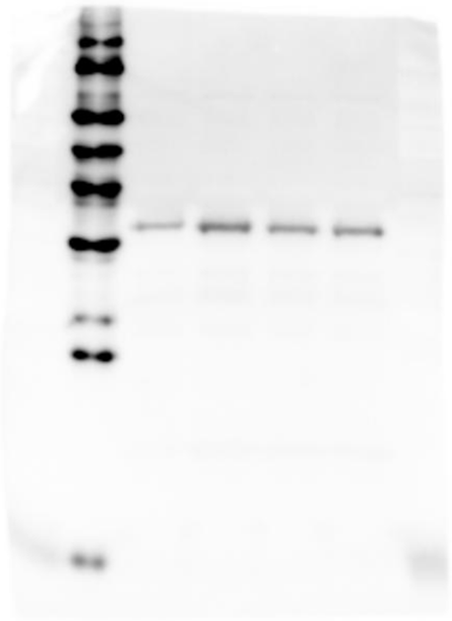

Fig5d α-SMA

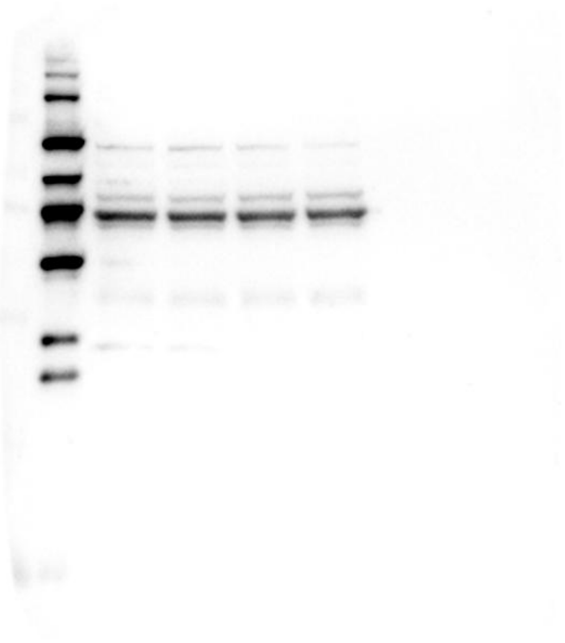

Fig5d β-Tubulin

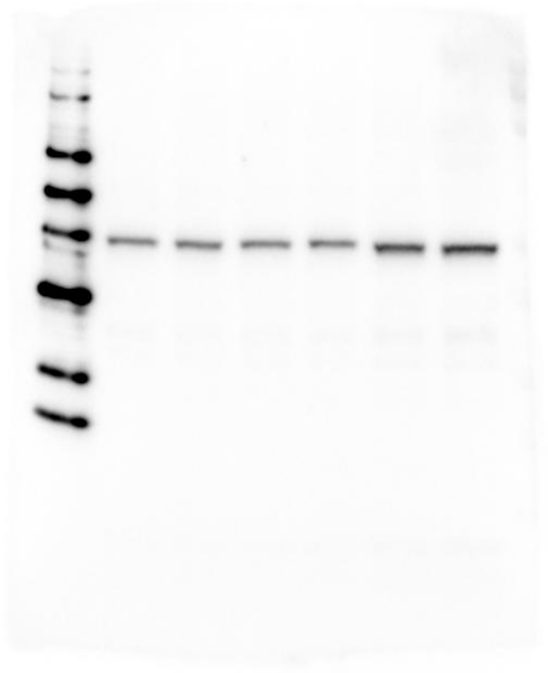

Fig6a p-Smad2

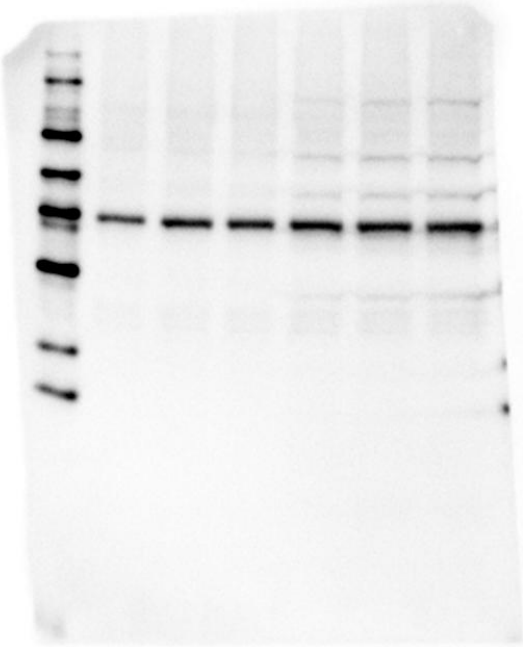

Fig6a p-Smad3

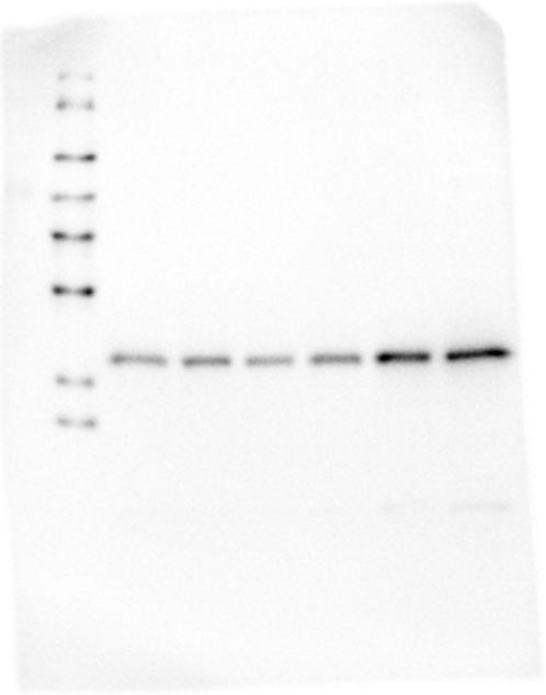

Fig6a SIP1

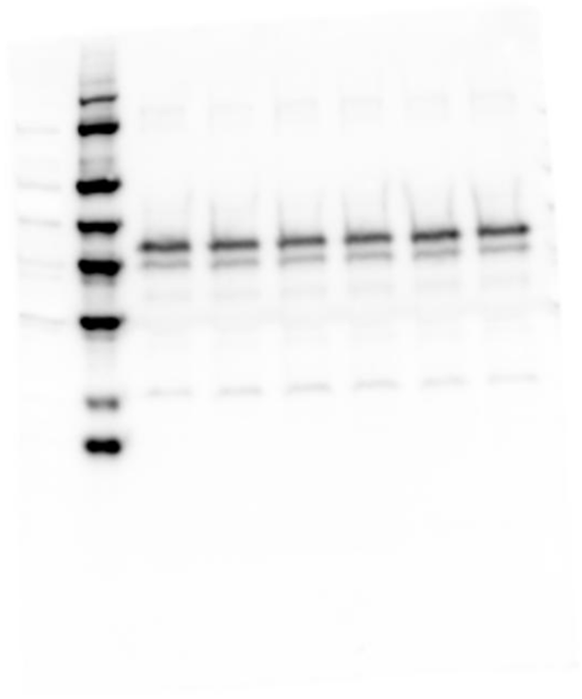

Fig6a Smad2/3

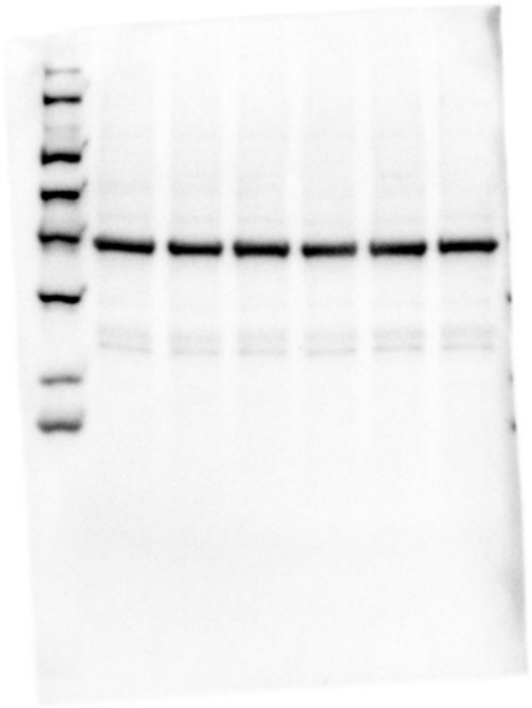

Fig6a β-Tubulin

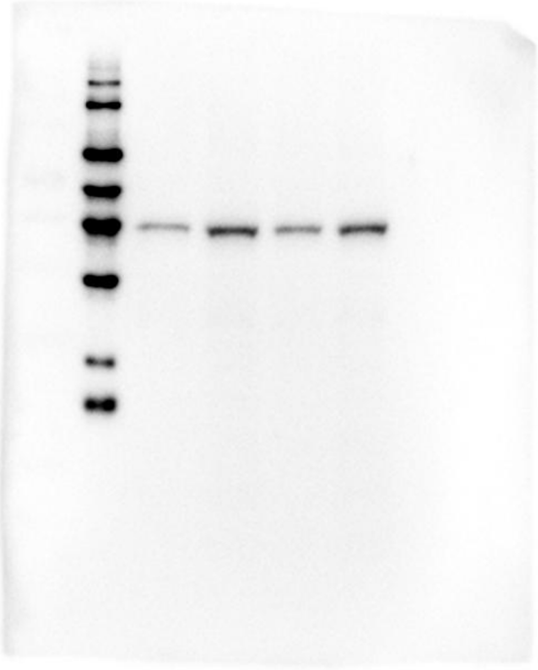

Fig6b p-Smad2

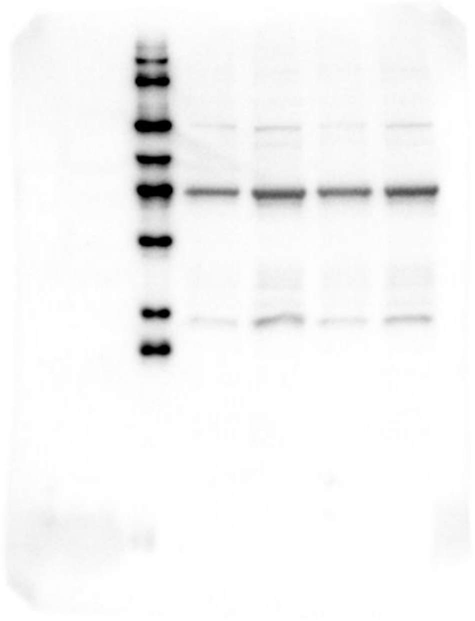

Fig6b p-Smad3

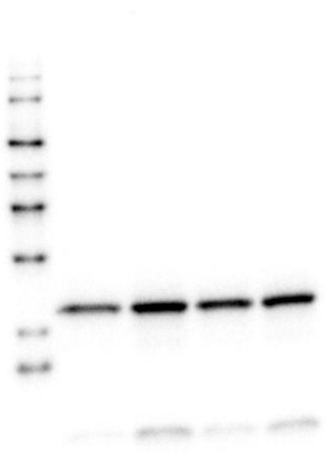

Fig6b SIP1

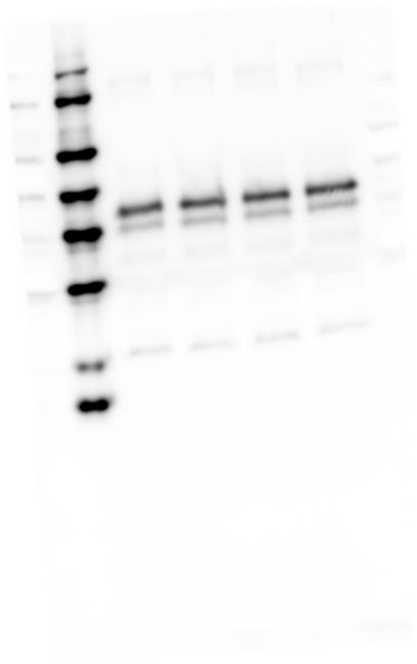

Fig6b Smad23

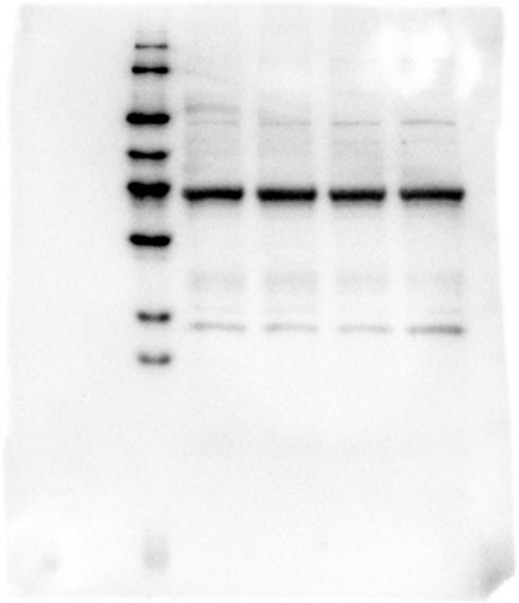

Fig6b β-Tubulin
